# Post-Transcriptional Regulation through Long Non-Coding RNAs (lncRNAs)

**DOI:** 10.3390/ncrna7020029

**Published:** 2021-04-29

**Authors:** Giuseppina Pisignano, Michael Ladomery

**Affiliations:** 1 Department of Biology and Biochemistry, University of Bath, Claverton Down, Bath BA2 7AY, UK; 2 Faculty of Health and Applied Sciences, University of the West of England, Coldharbour Lane, Frenchay, Bristol BS16 1QY, UK

The discovery of thousands of non-coding RNAs (ncRNAs) pervasively transcribed from the eukaryotic genome has revolutionized the “central dogma” of biology and shifted the attention on the role of RNAs as regulatory molecules, more than simply traditional mediators of genomic information. Non-coding RNAs are transcripts that do not encode proteins and are generally classified as short or long depending on their average size (< or >200 nt). Non-coding RNAs are found in nearly all organisms. Among them, the long non-coding RNAs (lncRNAs) play key roles in many biological processes in development and disease. Since their discovery, the lncRNA field has exploded, and new roles for lncRNAs are constantly emerging, making their investigation a priority in studying gene expression regulation at any level.

This Special Issue encases seven review papers and one original research article from experts in the ncRNA field and illustrates the main mechanisms through which lncRNAs modulate gene expression at the post-transcriptional level. This collection of articles provides a complete overview of their multifunctional roles and presents an additional layer of complexity in the regulation of gene expression and associated cellular processes.

LncRNA length, low expression, and lack of sequence conservation have frequently represented a major technical limitation in their identification and characterization. In their review, Carter et al. provide an exhaustive guide of both in silico and low-to-high throughput experimental approaches to assist researchers to face this challenge. They also offer critical insights to advance our understanding of how lncRNAs are involved in tumorigenesis [1].

A wide range of RNA-binding proteins (RBPs) have been shown to cooperate with lncRNAs to regulate gene expression. In their review, Briata and Gherzi draw attention to the complexity of lncRNA–RBP associations [2]. They illustrate the variety of mechanisms through which lncRNA–RBP complexes can control essentially all post-transcriptional processes in the cell. Sadeq et al. discuss how endogenous lncRNA-associated dsRNA structures are tolerated, whereas viral-derived dsRNA triggers a complex defence network; and further examine the potential implications in the context of autoimmune disease and cancer treatments [3].

In their review, Pisignano and Ladomery describe multiple mechanisms through which lncRNAs contribute to the regulation of alternative splicing and how their action further enhances the expression of mRNA-splicing variants, thereby increasing proteomic diversity in complex organisms [4].

In a more cytoplasmic context, Karakas and Ozpolat discuss how lncRNAs can affect mRNA translation by controlling translation factors and signalling pathways in normal and tumour conditions [5], while Sebastian-delaCruz et al. highlight the importance of lncRNAs in the regulation of mRNA stability and turnover as the basis for the correct functionality of cellular processes and homeostasis [6]. In this regard, in another work presented in this Special Issue, Munz et al. found in a diffuse, large B cell lymphoma cell line a lncRNA (*lncTNK2-2:1*) associated with the increased stability of transcripts that are affected by mTOR inhibition and responsible for the DNA damage response [7].

Fonouni-Farde et al. conclude this Special Issue by describing how plant lncRNAs use sophisticated mechanisms to regulate RNA degradation, alternative splicing, translation, post-translational modifications and even protein localisation [8].

Taken together, this Special Issue highlights the relevance of lncRNAs as crucial regulatory molecules in most post-transcriptional regulation mechanisms, both in animals and plants, and aims to encourage research groups and young researchers to further develop new studies in the field. A more comprehensive understanding of the molecular mechanisms of post-transcriptional regulation by lncRNAs will certainly advance our understanding of the many intricate cellular processes that are still far from being fully elucidated.

## References

[1] Carter J., Ang D., Sim N., Budiman A., Li Y. (2021). Approaches to Identify and Characterise the Post-Transcriptional Roles of lncRNAs in Cancer. Non-Coding RNA.

[2] Briata P., Gherzi R. (2020). Long Non-Coding RNA-Ribonucleoprotein Networks in the Post-Transcriptional Control of Gene Expression. Non-Coding RNA.

[3] Sadeq S., Al-Hashimi S., Cusack C., Werner A. (2021). Endogenous Double-Stranded RNA. Non-Coding RNA.

[4] Pisignano G., Ladomery M. (2021). Epigenetic Regulation of Alternative Splicing: How LncRNAs Tailor the Message. Non-Coding RNA.

[5] Karakas D., Ozpolat B. (2021). The Role of LncRNAs in Translation. Non-Coding RNA.

[6] Sebastian-delaCruz M., Gonzalez-Moro I., Olazagoitia-Garmendia A., Castellanos-Rubio A., Santin I. (2021). The Role of lncRNAs in Gene Expression Regulation through mRNA Stabilization. Non-Coding RNA.

[7] Munz N., Cascione L., Parmigiani L., Tarantelli C., Rinaldi A., Cmiljanovic N., Cmiljanovic V., Giugno R., Bertoni F., Napoli S. (2021). Exon–Intron Differential Analysis Reveals the Role of Competing Endogenous RNAs in Post-Transcriptional Regulation of Translation. Non-Coding RNA.

[8] Fonouni-Farde C., Ariel F., Crespi M. (2021). Plant Long Noncoding RNAs: New Players in the Field of Post-Transcriptional Regulations. Non-Coding RNA.

